# Neonatal donation: are newborns too young to be recognized?

**DOI:** 10.1007/s00431-021-04139-3

**Published:** 2021-06-09

**Authors:** Alicija Vileito, Christian V. Hulzebos, Mona C. Toet, Dyvonne H. Baptist, Eduard A. A. Verhagen, Marion J. Siebelink

**Affiliations:** 1 Department of Neonatology, Beatrix Children’s Hospital, University Medical Center Groningen, University of Groningen, P.O. Box 30.001, 9700 RB Groningen, the Netherlands; 2 Department of Neonatology, Wilhelmina Children’s Hospital, University Medical Center Utrecht, Utrecht, the Netherlands; 3 University Medical Center Groningen Transplant Center, University Medical Center Groningen, University of Groningen, Groningen, the Netherlands

**Keywords:** End-of-life care, Neonatal, Organ donation, Pediatric; Tissue donation

## Abstract

Neonatal organ and tissue donation is not common practice in the Netherlands. At the same time, there is a transplant waiting list for small size-matched organs and tissues. Multiple factors may contribute to low neonatal donation rates, including a lack of awareness of this option. This study provides insight into potential neonatal organ and tissue donors and reports on how many donors were actually reported to the procurement organization. We performed a retrospective analysis of the mortality database and medical records of two largest neonatal intensive care units (NICUs) in the Netherlands. This study reviewed records of neonates with a gestational age >37 weeks and weight >3000g who died in the period from January 1, 2005 through December 31, 2016. During the study period, 259 term-born neonates died in the two NICUs. In total, 132 neonates with general contra-indications for donation were excluded. The medical records of 127 neonates were examined for donation suitability. We identified five neonates with documented brain death who were not recognized as potential organ and/or tissue donors. Of the remaining neonates, 27 were found suitable for tissue donation. One potential tissue donor had been reported to the procurement organization. In three cases, the possibility of donation was brought up by parents.

*Conclusion*: A low proportion (2%) of neonates who died in the NICUs were found suitable for organ donation, and a higher proportion (12%) were found suitable for tissue donation. We suggest that increased awareness concerning the possibility of neonatal donation would likely increase the identification of potential neonatal donors.

**Table:** 

**What is Known:** • *There is an urgent need for very small organs and tissues from neonatal donors* **What is New:** • *A number of neonates who died in the NICU were suitable organ or/and tissue donors but were not recognized as donors.* • *Knowledge on neonatal donation possibilities is also important for proper counseling of parents who sometimes inquire for the possibility of organ and tissue donation.*

**What is Known:**

• *There is an urgent need for very small organs and tissues from neonatal donors*

**What is New:**

• *A number of neonates who died in the NICU were suitable organ or/and tissue donors but were not recognized as donors.*

• *Knowledge on neonatal donation possibilities is also important for proper counseling of parents who sometimes inquire for the possibility of organ and tissue donation.*

## Introduction

The rate of organ donation in neonates is at least 10 times lower than it is among older infants [1]. For decades, there has been a shortage of organs and tissues for pediatric and neonatal transplantation [2]. The transplant waiting list of the Eurotransplant registry, which covers eight countries, includes a substantial number of infants aged 0–1 year. Small, size-matched organs or tissues—mainly the liver, lungs or heart can be obtained from neonatal donors [2]. Recently, De Luca and colleagues published results of a survey among members of the European Society for Pediatric and Neonatal Intensive Care (ESPNIC) on lung transplantation in neonates and in infants: a limited donors’ availability appeared to be the main obstacle for lung transplantation [3]. The shortage of organs results in waiting list mortality [4, 5]. It is reasonable to assume that reducing time on the waiting list will reduce waiting-list mortality and, conversely, improve growth and neuro-developmental outcomes of young transplant patients. This raises the questions of whether and how waiting list mortality could be reduced for neonatal and small pediatric patients. In addition to the surgical reduction of the size of donor organs, the early identification of succumbing newborn infants who may potentially serve as neonatal donors is imperative. The latter has been suggested by Bratton et al. as a strategy for augmenting pediatric organ donation [6]. Siebelink et al. have shown that approximately 10% of the patients dying in the pediatric intensive care unit (PICU) were correctly identified as potential organ donors, older children were over-represented in comparison with younger potential donor candidates [7]. In addition to young infants who die in a PICU, patients dying in a neonatal intensive care unit (NICU) could be appropriate candidates for the donation of small-sized organs. This possibility is particularly relevant to the critical needs of small pediatric patients and neonates on the transplant waiting list.

In current practice, neonatal donation is usually not considered, and less than 10% are actually referred to organ procurement organizations before withdrawal of life-sustaining treatment in a NICU [8]. This situation could be attributed, at least in part, to insufficient knowledge about the rules for donation and the corresponding legislation, a lack of institutional donation protocols, and the relative rarity of donation in neonates [7, 9–11]. In addition, neonatal donation is frequently judged as contra-indicated, based on perceived multi-organ injury, as it is likely to occur after profound perinatal asphyxia. Furthermore, the very strict criteria for declaring brain death in neonates*,* including the obliged repeated examination after an observation period of 24–48 h, make donation after brain death (DBD) difficult, or even impossible [12]. According to the current Dutch Organ Donation Act protocol, organ donation after circulatory death (DCD) is not possible at this age [13]. In the Netherlands, the neonatologist in the NICU is responsible for discussing the possibility of donation with parents of potential donors and approaching them for consent for organ or tissue donation. After donation consent has been obtained, the potential donor is reported to the Dutch organ procurement organization which determines suitability for organ and tissue donation and proceeds with the donation process. In the Netherlands, a substantial proportion of neonates who have poor prognoses but who, in most cases, are not brain dead, die following the careful decision of their medical team and parents to withdraw life-sustaining treatment [14, 15]. If considered and discussed with parents in a timely manner, organ donation by these patients may offer opportunities for increasing neonatal donor potential. Assessing the feasibility of this would obviously require insight into the number of potential neonatal donors, the number who are likely to have been missed, and the number who have actually been reported to the procurement organization. Therefore, we designed a study in two NICUs in the Netherlands.

## Materials and methods

We performed a retrospective study in two large NICUs in the Netherlands (i.e., in the University Medical Centers of Utrecht and Groningen). We selected these NICUs because, at that time, they were the largest NICUs in the Netherlands, with a total admission capacity of 48 newborn infants. On average both NICUs admitted 500 infants each year. Approximately 50% were preterm infants with a gestational age less than 32 weeks. The remainder were (late) preterm infants or term infants with gastro-intestinal, cardiovascular, or neurological disorders. Both NICUs received out of hospital transfers for whole body therapeutic hypothermia of infants with hypoxic ischemic encephalopathy after perinatal asphyxia.

We analyzed medical records from January 1, 2005 through December 31, 2016 to answer the following questions:

1. How many neonates could have been identified as potential organ and/or tissue donors?
2. How many neonates were actually identified as potential donors?
3. How often was donation discussed with parents?
4. How many times was donation actually effectuated?

To identify deceased neonates, we queried both the databases of the individual hospitals and the database of the national Neonatal Intensive Care Evaluation (NICE). Demographic data and NICU admission data were retrieved from the hospital databases. Data included the gestational age, sex, weight, admission diagnosis, cause of death, and postnatal age of death. The analysis followed the protocol of the Dutch Organ Donation Act and included the investigator’s assessment of the suitability of the deceased patients for organ and/or tissue donation and whether the consultant neonatologist had discussed the possibility of donation with the parents.

According to the current Dutch Organ Donation Act, a neonate is eligible for organ and tissue donation when the gestational age is >37 weeks and the actual weight is >3000g [13]. In addition to these criteria, brain death is an essential prerequisite for DBD. The formal diagnosis of brain death requires repeated neurological examination. For neonates less than 7 days old, a neurological reexamination is required after an observation period of 48 h. For patients older than 7 days, but younger than 2 months of age, an observation period of 24 h is required. From birth infants can donate heart, lungs, and kidneys, with liver donation being possible starting at 1 month of age. Regarding tissue donation, the protocol allows only heart-valve donations from infants.

In accordance with the research protocol, our study began with the identification of all neonates who had died in the two NICUs with a gestational age >37 weeks and weight >3000g, in the NICE database. We reviewed NICE records of each of these patients and then excluded all neonates with general contra-indications for donation, as presented in Table 1. Then we retrieved medical records of the included patients and analyzed them for suitability for organ and/or tissue donation. Decisions concerning suitability for organ and/or tissue donation were based on records in medical files, including diagnoses (and documentation of brain death).

**Table 1:** General contra-indications for donation in the Netherlands

General contra-indications for donation	
Multisystem disease (mitochondrial/metabolic/chromosomal)	
Congenital heart disease	
Multiple congenital anomalies	
Active infection, sepsis	
Hematological malignancy or solid tumor	

Medical records were analyzed by two investigators (AV and MS) with extensive experience in pediatric donation. As a quality check, 10% of the medical records were also analyzed by an independent clinical research coordinator (DB). There was 100% agreement between all assessors. The data were analyzed in SPSS 22.0 (IBM Corp, New York, USA) and Excel.

The study complied with the national regulations concerning privacy and medical research. According to the Dutch law, survey studies like this one do not require approval by a medical ethical review board. This was confirmed for the present study by the Medical Ethical Review Board of the University Medical Center Groningen (M17.213355).

## Results

**Fig. 1:**
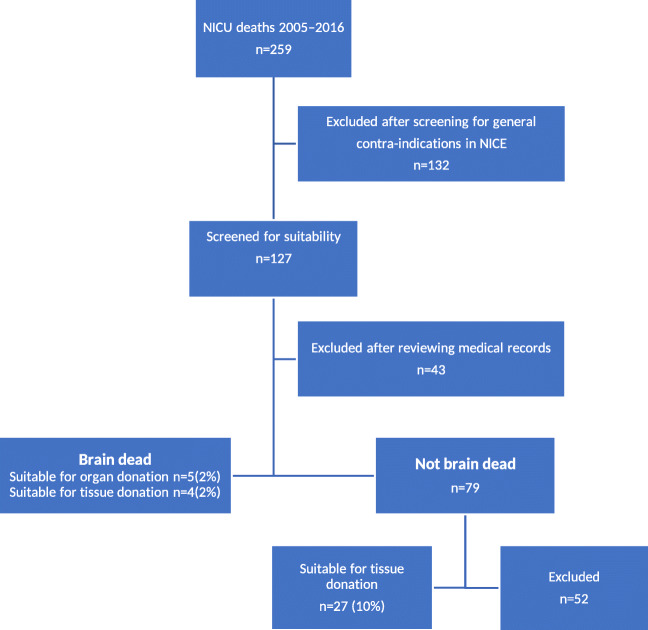
Flowchart for potential neonatal donors

During the 11-year study period, 259 term neonates died in the two units (24 deaths per year). Characteristics of these neonates are presented in Table 2. We excluded 132 neonates after initial screening NICE database records for general contra-indications (Fig. 1). Of the excluded neonates, 115 had single or multiple congenital disorders, such as: chromosomal anomalies, mitochondrial/metabolic conditions, congenital heart disease, or other (multiple) congenital anomalies. In addition, 15 neonates had been diagnosed with sepsis or active infection, and two had tumors (Table 3). The medical records of the remaining 127 neonates were closely screened for donation suitability. After analysis of the medical records, 43 neonates were excluded from the potential donor list: one neonate had an active infection, one had multiple congenital anomalies, five had been diagnosed with mitochondrial/metabolic conditions, 30 had been diagnosed with multi-organ failure, two had unknown diagnoses, and the medical records of four neonates could not be assessed.

**Table 2:** Characteristics of term-born neonates who died in the NICUs during the study period

Characteristics	NICU 1	NICU 2
N	101	158
Median gestational age, (weeks + ^d/7^)	39^+3/7^	39^+3/7^
Mean/maximum weight (g)	3700/4980	3605/5100
Male/female, n (%)	56 (55)/46 (45)	88 (56)/70 (44)

^d^day

**Table 3:** Neonates excluded based on general contra-indications

General contra-indication n (%)	NICU 1 (n=101)	NICU 2 (n=158)	Total (n=259)
Mitochondrial/metabolic conditions	4 (4)	9 (6)	13 (5)
Congenital heart diseases	24 (24)	29 (18)	54 (20)
Chromosomal anomalies	5 (5)	14 (9)	19 (7)
Other multiple congenital anomalies	12 (12)	18(11)	29 (11)
Sepsis/infection	5 (5)	10 (6)	15 (6)
Tumor	2 (2)	-	2 (%)
Total excluded	52 (51)	80 (50)	132 (51)

Of the 84 neonates without medical contra-indications, five were diagnosed as being brain dead. Demographic characteristics of potential organ donors are presented in Table 4. Of the five patients who had been diagnosed as brain dead, only two were suitable organ or tissue donors. Those two neonates were potential heart, kidney, or heart-valve donors. Neither of these children were recognized as potential organ or tissue donors, and the donation options were therefore not discussed with their parents. Three other brain-dead neonates were considered unsuitable for organ donation due to persistent circulatory insufficiency. Two of these patients were potential heart-valve donors, but they were not recognized as such, and tissue donation was not discussed with their parents. The medical records of the remaining patient did not provide any information on the anatomical structure of the heart. We were therefore unable to establish suitability for tissue donation.

**Table 4:** Demographic characteristics of potential organ donors

Potential organ donors	n=5
NICU1	3
NICU2	2
Male	2
Female	3
Age at admission (days)	0 (0–1)
Age at death(days)	2 (1–3)
Gestational age, weeks + ^d/7^	39^+1/7^
Weight median (IQR), in grams	3575 (3240–4090)
Diagnosis on admission:	
Perinatal asphyxia	5

^d^days.

Of 79 possible tissue donors, 27 neonates were assessed as suitable for tissue donation. The medical records of 51 neonates did not provide any information on the anatomical structure of the heart, and we were therefore unable to establish suitability for heart-valve donation. One patient was not suitable for heart-valve donation due to a ventricular septal defect.

Of all 27 suitable tissue donors, the possibility of tissue donation was discussed in only three cases. In each of these cases, the parents inquired about this possibility. Medical staff had not recognized two of the potential tissue donors, and they were therefore not reported to the procurement organization. Only one potential tissue donor was recognized and reported to the procurement organization. Due to logistical reasons, donation was not effectuated.

## Discussion

This study describes the potential of neonatal donors in two large NICUs in the Netherlands. The main questions concerned how many neonates were potential organ and/or tissue donors, how many have been missed, and how many were actually reported to the Dutch organ and tissue procurement organization.

Our results indicate that, of the neonates who died in the two NICUs during the period of study, 2% were potential organ donors and 12% were potential tissue donors. The corresponding percentages for all PICUs in the Netherlands were higher (potential organ donors: 11%; potential tissue donors: 19%) [7]. This is not surprising, given current age and weight restrictions [13]. We have no clear explanation for why the percentages identified in this study are lower than the percentage (13%) of potential neonatal DBD donors in the UK [16]. Our results nevertheless suggest that a small proportion of neonates who died in two NICUs could have been potential organ and/or tissue donors. Extrapolation of these figures to all nine NICUs in the Netherlands suggests that there are approximately two or three potential neonatal organ donors per year in the Netherlands.

The pediatric transplant waiting list reflects an urgent need for very small organs and tissues, and especially for hearts, livers, and small heart valves. Children die while on the waiting list due to the shortage of such small-sized organs and tissues. Despite the small absolute numbers, it could be argued that every donor in a NICU could save the life of an infant in need of transplantation. As an example, heart transplantation performed during the neonatal period is a durable therapy for infants with congenital heart disease, and associated with a minimal need for re-intervention [17]. However, the primary limiting factor is the shortage of donors [17–19]. The same holds true for lung transplantation in young infants with surfactant dysfunction disorders and congenital diseases [5, 20, 21]. Although showing promising results, neonatal lung transplantation is extremely rare and mostly performed in North America [5, 20, 21]. The ESPNIC expressed the need to develop a European network for lung or lung and heart transplantations for young infants to improve outcome of such a transplantation and accumulate experience in Europe [3]. Experience is advancing with regard to renal transplantation in children. En-bloc renal transplantation from young donors is acceptable and safe, with a low complication rate, also in pediatric recipients [22]. Similarly, several studies have suggested that the utility of livers from donors with low body weight could be a potential strategy for increasing the availability of donors for well-selected pediatric recipients [23, 24]. Moreover, kidney and liver transplantation show good results even with size and/or weight mismatched organs [22–24]. Organ size matching is more delicate for heart and lung transplantation [18, 21]. Despite surgical organ-specific technical challenges, every neonatal donor could reduce waiting list mortality of small pediatric recipients. For proper counseling of parents and early and successful identification of possible neonatal donors, timely consulting of an organ procurement organization, that has knowledge on various aspects of neonatal donation and transplantation, is essential.

As indicated by our results, quite a number of neonates were potential heart-valve donors, but medical professionals did not identify potential tissue donors until after the possibility was raised by parents. Although in general, there are less opportunities for neonatal donation when compared to older infants, there seem to be more opportunities to discuss neonatal donation with parents. This ‘lack of awareness’ is also described in a case study by Kieboom et al., who conclude that several donation opportunities were missed as a result [25]. Lack of awareness can be caused by a lack of experience with donation and/or a lack of knowledge about the possibility of neonatal donation [26, 27]. Limited data on neonatal donation makes it difficult to establish reasons for the low rate of donors’ identification and referral rates among neonates [28]. One particularly interesting finding of our study is that discussions about donation were initiated by the parents of potential donors. This might indicate that parents expect donation to be a part of palliative and end-of-life care in the NICU. Few studies have stated that donation could help the parents to cope with their loss and help them to find meaning in the death of their child [22, 29]. In particular, it is important to understand the value of discussing the possibility of donation with parents before death of the infant, in order to address their assumptions regarding donation [30]. It is important to increase awareness among NICU professionals that donation can be a part of palliative and end-of-life care in the NICU [26]. This corresponds to the call of Mathur et al. for palliative care protocols for potential donors in the NICU [31]. Furthermore, neonatal donation could arguably be regarded as a logical component of a comprehensive pediatric donation protocol [26]. However, the efficacy of such a national pediatric donation protocol remains currently unknown [28].

Despite the small number of potential neonatal donors in NICUs in the Netherlands, we can conclude that every recognized donor whose donation could be effectuated could have a significant impact on waiting list mortality for this specific age group. This raises questions concerning how can we ensure that neonatal donors are not too young to be recognized and which obstacles are standing in the way of this endeavor.

We believe that the existing Dutch donation protocol is not designed specifically for neonates and infants, and it limits the identification of potential donors in the NICU. The protocol lacks specific lower age and weight donation criteria. The formal process for determining brain death, as specified in the protocol, is complicated [13]. A declaration of brain death in neonates and infants requires repeated examination after an observation period of 24–48 h [13]. To our knowledge, countries differ in this regard. Although brain death is not exceptional in the neonatal population, the determination of this diagnosis is difficult [12]. A long observation period before final declaration of brain death could impose an excessive burden on parents in these circumstances. As concluded by few authors, the low rate of neonatal donation could be due to the complexity of diagnosing brain death in such young patients [1, 16]. The possibility of DCD may also increase neonatal donation rates. Theoretically, if all potential tissue donors would be potential DCD organ donors, the percentage of organ donation would increase up to 12 %. Current regulation on neonatal donation limits to DBD in the Netherlands, but this varies per country [13, 16, 28, 29, 32, 33]. For example, DCD and DBD is possible in Spain and the UK in neonates of 34 or 37 weeks’ gestation age, respectively, and even donation in an anencephalic newborns is considered feasible [16, 28, 29, 32]. This is in contrast with strict Swiss legal and ethical restrictions, which make neonatal donation impossible [33].

Several limitations to this study should be mentioned. Due to its retrospective character, it was impossible to conduct a complete assessment of all medical records. In some cases, the records did not contain sufficient information to establish the patient’s suitability for organ and/or tissue donation. A prospective, observational study of neonates dying in the NICU would be necessary in order to generate more information about donor potential and actual practice in the determination of brain death in NICUs.

In conclusion, we found that only a few of the neonates who had died in the NICU were potential organ donors. This figure was higher for potential tissue donors. Early recognition of potential donors among neonates dying in the NICU could expand the donor pool and benefit children on the waiting list [22]. Medical professionals apparently have little awareness concerning the possibilities of donation in the NICU and parents, not medical staff, inquire about the possibility of donation. This situation should be improved. The formulation of a specific pediatric donation protocol that includes procedures for neonatal organ and tissue donation is an essential imperative, as is the reconsideration of the criteria for brain death in infants. In our opinion, any donor—no matter how small—is worth recognition and could potentially save a life.

## Data Availability

Data is available on request from first author.

## Abbreviations

DBD: Donation after brain death
DCD: Donation after circulatory death
ESPNIC: European Society for Pediatric and Neonatal Intensive Care
NICE: Neonatal intensive care evaluation
NICU: Neonatal intensive care unit
PICU: Pediatric intensive care unit

## Code availability

N/A
